# The Importance of Developing and Standardizing Gamete, Embryo and Larvae Handling in Aquatic Animals

**DOI:** 10.3390/ani13020270

**Published:** 2023-01-12

**Authors:** Estefanía Paredes, Victor Gallego

**Affiliations:** 1 Grupo ECOCOST, Centro de Investigación Mariña (CIM), Departamento de Ecoloxia e Bioloxía Animal, Universidade de Vigo, 36310 Vigo, Spain; 2 Center of Marine Sciences (CCMAR), Universidade do Algarve, 8005-139 Faro, Portugal

Artificial reproduction in aquatic animals usually involves the collection and handling of gametes both from males and females in a way that secures their quality and optimizes the fertilization event. There are innumerable specie- and family-specific protocols among the various aquatic species that inhabit bodies of water, from freshwater lagoons to seawater oceans. In that sense, this Special Issue aims to gather high-quality papers addressing different areas, focusing on gamete collection and handling, gamete storage (both short- and long-term storage), in vitro fertilization techniques, embryo development studies and the larvae management of different aquatic animals.

Whether our final goal is the advancement of basic research, aquaculture exploitation or biodiversity conservation, exploring aquatic organism’s factors, such as seasonal spawning, type and strategy of reproduction, spawning induction, gamete extraction, fertilization factors, quality of the gametes and proper incubation of the larvae, is a key factor to enabling our studies [1,2]. Due to their variability, factors between taxa and even species-specific methods need to be developed and shared in the scientific community.

When dealing with sessile organisms that present external fertilization, such as oysters (*Crassostrea virginica* [3]) or clams (*Mesodesma donacium* [4]), animals do not always have to be induced to spawn. Mature gonads can sometimes be stripped and the gametes then collected directly, such as in the case of oysters, although there are not always species that present smaller sizes or internal fertilization. The spawning of clams or mussels has to be induced, for which several spawning inductors have been used over the years, from overfeeding to tide simulation or temperature shocks [5]. In all cases, protocols need to be established, shared and standardized within the scientific community to successfully obtain gametes from different mollusks, as well as sea urchins, which also present with external fertilization. Sea urchins are model organisms and, therefore, some species of sea urchins, such as *Strongylocentrotus purpuratus* or *Paracentrotus lividus,* are quite well studied and have many protocols in place; however, this is not the case for other sea urchins [6], as it has been shown in many cases that protocols would have to be adapted to particular species. Important knowledge about reproduction is not often well-known or standardized, such as contact time, the egg:sperm ratio, optimum parameters for embryo incubation and expected outcome after larval rearing, but is key information for marine invertebrates [2,6].

When dealing with marine and freshwater fish, there is also a wide diversity of reproductive strategies [7]. Most of them share external fertilization, where gametes from both males and females are released into the aquatic environment. These gametes could be crucial factors for the achievement of successful fertilization and hatching rates throughout in vitro fertilization trials [1]. For releasing gametes, fish broodstock must be properly matured, and some environmental factors, such as salinity, photoperiod and/or temperature, can stimulate the sperm and oocyte production [8]. In many other cases, these factors are not optimal, and it is necessary to apply short or long hormonal treatments in species with reproductive bottlenecks [9,10]. Once male and female gametes are collected, it is essential to keep their quality using extenders at different ratios [11] and/or adding antibiotics in fertilization trials. Finally, the choice of the egg:sperm ratio is also an essential factor that needs to be considered for the standardization and optimization of in vitro fertilization trials on the aquaculture sector. Generally, an excess of sperm is used in in vitro fertilization trials both in freshwater and seawater species, but an appropriate combination of the number of spermatozoa per oocyte must be used in order to enhance the reproductive efficiency in fish farms and to avoid wasting sperm when available gametes are limited [12].

Beyond theoretical considerations, an emphasis has been placed on a wide range of factors studied to determine proper protocols for handling, spawning and reproducing a wide range of different species in different taxa. Further investigation is required to refine the protocols and to extend them to other species whenever possible. Nonetheless, the contributions of this Special Issue are hoped to provide further insights regarding these different aspects, while promoting novel investigations targeting the standardized and successful reproduction of aquatic animals.
